# Aya Contigo: evaluation of a digital intervention to support self-managed medication abortion in Venezuela

**DOI:** 10.3389/fgwh.2024.1401779

**Published:** 2024-10-17

**Authors:** Kathryn Cleverley, Anjali Sergeant, Nina Zamberlin, Susana Medina, Genevieve Tam, Roopan Gill

**Affiliations:** ^1^ Department of Family Medicine, Queen’s University, Kingston, ON, Canada; ^2^ Department of Medicine, University of British Columbia, Vancouver, BC, Canada; ^3^ Fòs Feminista, New York, NY, United States; ^4^ Vitala Global, Vancouver, BC, Canada; ^5^ Department of Obstetrics and Gynecology, University of Toronto, Toronto, ON, Canada

**Keywords:** medication abortion, contraception, sexual and reproductive health (SRH), global women’s health, digital health, human-centered design

## Abstract

**Background:**

Venezuela continues to face a humanitarian crisis, where healthcare is difficult to access and abortion is legally restricted. In response to a growing need for life-saving abortion and sexual and reproductive health (SRH) services, a digital application called Aya Contigo was co-developed with local partners to support self-managed medication abortion. We sought to evaluate this digital health tool among pregnant people seeking abortion in Venezuela.

**Methods:**

This is a mixed-methods pilot evaluation of Aya Contigo, a digital tool for pregnant people seeking abortion in Venezuela. From April to June of 2021, people in the first trimester of pregnancy were recruited via passive sampling. Once enrolled, participants accessed information and resources on the application and were supported by study team members over an encrypted chat. Following medication abortion, participants completed an online survey and a semi-structured interview. Descriptive statistics were used to evaluate the survey responses. Interviews were coded thematically and analyzed qualitatively with NVivo.

**Results:**

Forty participants seeking medication abortion in Venezuela were recruited to the study and given access to Aya Contigo. Seventeen completed the online survey (42.5%), with all participants identifying as women and a mean age of 28 (range 19–38; SD 5.55). Participants expressed confidence in Aya Contigo; 53% (9/17) felt “very supported” and the remaining 47% (8/17) felt “somewhat supported” by the app throughout the self-managed abortion process. The app was rated as highly usable, with an overall System Usability Scale score of 83.4/100. Thirteen respondents participated in a semi-structured phone interview, and qualitative analysis identified key themes relating to the experience of seeking abortion in Venezuela, the user experience with Aya Contigo, and the app's role in the existing ecosystem of abortion and contraceptive care in Venezuela.

**Discussion:**

This mixed-methods pilot study demonstrates that the Aya Contigo mobile application may support pregnant people seeking medication abortion and post-abortion contraceptive services in Venezuela. Participants valued the provision of evidence-based information, virtual accompaniment services, and locally-available sexual and reproductive health resources via the digital tool. Further research and interventions are needed to ensure that all pregnant people in Venezuela can access safe abortion and contraceptive resources.

## Introduction

For over a decade, the population of the Bolivarian Republic of Venezuela has faced a humanitarian crisis stemming from highly precarious political and socio-economic conditions (1). While Venezuela was once an oil-rich economy, the succession of authoritarian socialist governments and foreign sanctions provoked financial collapse in 2012, and the country has not recovered since (2). In fact, severe hyperinflation and plummeting wages have resulted in crippling poverty for nearly ninety percent of citizens (1). Though the Venezuelan government has not disclosed epidemiologic data since 2017, the 2022–2023 United Nations Humanitarian Response Plan for Venezuela estimates that over 5.2 million people face difficulty accessing basic necessities such as food, clean drinking water, and health services (3). This has resulted in the migration of over seven million Venezuelans to nearby Latin American countries and beyond. In the midst of a massive refugee crisis facing the region, those who remain in Venezuela are often overlooked (1).

Girls, women, and people who can become pregnant are disproportionally affected by humanitarian crises, where access to sexual and reproductive health (SRH) services is often limited or non-existent (4, 5). In Venezuela, there is an extreme scarcity of available SRH resources, including contraceptive options and abortion services. Access to medical and surgical abortion is highly restricted and stigmatized as abortion is illegal in Venezuela except to save the life of the pregnant woman (6–8). In addition to the legal restrictions, medications such as mifepristone and misoprostol that can induce abortion are prohibitively expensive (7, 9). In this context, abortions still occur regularly, often through unsafe means (9–11). Maternal mortality rates are rising in Venezuela, having increased from 126 maternal deaths per 100,000 live births in 2015 to 259 maternal deaths in 2020 as estimated by the World Health Organization (WHO) (12). Venezuela has the highest maternal mortality rate in the Americas by a significant margin, with likely over ten percent of maternal deaths associated with unsafe abortions (13).

Self-managed medication abortion is a safe and effective means of terminating pregnancy in the first trimester (14–16). As the WHO describes, self-managed medication abortion is managed by the pregnant person either partially or completely outside of a healthcare facility (17). In many Latin American countries with legal restrictions around abortion, local organizations have supported pregnant people in self-managed medication abortion for over two decades (18). In this harm-reduction focused approach, organizations may offer evidence-based information, emotional and medical support, and essential abortion medication to pregnant people. Often, this involves the direct support of “accompaniers,” who are trained to guide people through the medication abortion process (19). Self-managed medication abortion may be the preferred choice for abortion for pregnant people, as the process enables discretion and can empower individuals to be active agents in their own healthcare (20, 21). In legally restrictive and low-resource settings such as Venezuela, this method may be the only viable option for termination of a pregnancy.

Feminist and activist-led organizations in Latin America have been foundational in the sexual and reproductive rights movement in the region, crucially offering access to safe and effective abortion care (19, 22, 23). In Venezuela, sexual and reproductive health organizations continue to provide life-saving services despite limited resources and the risks of facing legal repercussions (9). Given ongoing structural challenges, there is a need for innovative solutions to expand access to self-managed medication abortion services amidst the Venezuelan humanitarian crisis, particularly for those facing socioeconomic precarity and/or living in rural or remote regions.

Digital health interventions including mobile applications, web-based tools, and telemedicine hold the potential to promote sexual and reproductive healthcare in under-resourced settings (24–28). In collaboration with local SRH organizations in Venezuela, a mobile application called Aya Contigo was developed with the goal of facilitating self-managed medication abortion and contraceptive care. The development process involved an in-depth contextual analysis that employed a human-centered design (HCD) approach (9). Venezuelan citizens, reproductive health experts, and key local and international abortion harm-reduction organizations were consulted during the tool's design and implementation phases. To our knowledge, this is the first digital health intervention for abortion and SRH in South America that utilizes an HCD approach (29).

As part of Aya Contigo's implementation in Venezuela, we conducted a mixed-methods evaluation of the application's ability to support self-managed medication abortion and post-abortion care in the first trimester. Our primary study objective is focused on evaluating Aya Contigo as a mobile application for medication abortion and contraceptive care within the Venezuelan context. Specifically, this study assesses the acceptability, safety, and efficacy of Aya Contigo among pregnant people seeking abortion services within a legally-restrictive and humanitarian setting. Our secondary objectives will support the study's primary aim by (1) describing the local context for people seeking abortion in Venezuela and (2) determining how Aya Contigo fits within the already-existing ecosystem of abortion care in Venezuela. Our findings will inform the iterative development of Aya Contigo and offer insights into digital solutions designed to improve access to abortion care in legally-restrictive and humanitarian settings.

## Materials and methods

### Overview

We present a mixed-methods pilot study to evaluate Aya Contigo, a mobile application developed by Vitala Global Foundation in partnership with local organizations and stakeholders in Venezuela. Vitala Global is a Canadian non-profit that co-creates and implements digital tools to support SRH needs (30). The application was developed with the principles of HCD, a method to create acceptable and feasible digital interventions with the potential to be scaled up sustainably (31–33). This model involves a participatory process that focuses on the needs and desires of end-users and relies on their input throughout the entire development cycle (31). A design-thinking approach holds the potential to reshape healthcare provision, particularly in contexts in need of innovative solutions (33). While HCD has been used to develop global health interventions such as clinical decision-making tools and patient-facing websites, few studies have adequately reported the methodologies and impacts of such interventions (32).

In order to understand the local needs of individuals seeking abortion in Venezuela, a contextual analysis was conducted to inform the development of Aya Contigo. As described elsewhere, this involved an in-depth desk review and stakeholder interviews focused on the legislation, policies, and practices that create barriers to abortion and SRH care in Venezuela (9, 11, 27). This user-centred process formed the basis for Aya Contigo, which was developed iteratively with community stakeholder involvement throughout (8).

Aya Contigo is a digital application that guides users through steps to self-manage their abortion and offers a personalized contraception decision-making tool. In order to protect privacy and security, each user is provided with a privacy statement that includes information on managing data stored on personal devices. The application's securitized back-end never collects any personally-identifiable user information and the limited aggregate data collected is stored on an encrypted and remote server and deleted permanently after a 3-month period. Data is collected for the purpose of periodic quality and impact assessments. Information collected is limited to the number of application downloads, the number of participants who opt-in to notifications, time spent engaging with the tool, and the most visited sites within the tool (i.e., the contraceptive method guide, the abortion completion checklist). This data is never sold to third parties.

Application users respond to questions that assess eligibility for medication abortion, including a gestational age calculation (based on last menstrual period and/or ultrasound dating calculations) and any contraindications to the abortion medication(s). The subsequent sections in the mobile application provide clear and evidence-based information on medication selection, dosage, and timing. Aya Contigo educates users on common medication abortion side effects and how to manage them, outlining self-care options and offering guidance on when to present to a healthcare facility. Once the user has followed the abortion medication regimen, the app offers a self-assessment tool for abortion completion based on current symptoms (Supplement A: Abortion Completion Checklist). It also includes a section on contraception that asks users about their sexual health preferences and possible contraindications to certain types of contraceptives. Based on the user's responses, Aya Contigo then provides an individualized set of evidence-based contraceptive methods.

### Participants

Study participants were recruited passively through online advertisements, sexual health and reproductive health clinics in Venezuela, and local and international abortion harm-reduction organizations. Participants aged 14 years and older who were located in Venezuela, pregnant and in the first trimester, interested in medication abortion, literate in Spanish (including the ability to read and understand the mobile application and study information), and who could access a functioning smartphone were included. Individuals were included if they were within the first trimester of pregnancy, defined as up to 12 weeks of gestation. If a person's pregnancy was beyond the first trimester, they were excluded from the study, in order to align with the current WHO guidelines and evidence base that supports the safety and efficacy of self-managed medical abortion within the first trimester (16, 34). Those who were unable to provide informed consent or participate in the study procedures were also excluded.

### Study design

In this mixed-methods study, we conducted online quantitative surveys and qualitative semi-structured telephone interviews from April 1st to June 15th, 2021. There is a need for descriptive data in the context of people seeking abortion in Venezuela, where little research has been possible in recent years due to the ongoing crisis. The study objectives are well-suited to include a qualitative component, as this approach enables an in-depth assessment of both local context and stakeholders’ individual perceptions and unique experiences (35). The online survey and qualitative interview guide were developed with multiple rounds of feedback from a multidisciplinary team of local and international experts and stakeholders. Interviews were conducted by IP and GL, two female and Spanish-speaking study team members from Venezuela with qualitative research training. To ensure high-quality research methods and data analysis, we adhered to the guidelines of the Consolidated Criteria for Reporting Qualitative Research (CORE-Q) checklist (36). While participant involvement in the dissemination of results was originally planned, security protocols limited communication with participants after study completion due to the legal restrictions and risks to those who choose to pursue abortion in Venezuela.

### Data collection

Individuals interested in study participation contacted the research team over WhatsApp via the number provided through partner organizations and online advertisements. Eligible participants reviewed information on the mobile health intervention and provided written informed consent via a signed digital form sent through the encrypted WhatsApp chat for the quantitative component of the study. Once enrolled, participants received a link and instructions to access and utilize the Aya Contigo application. The research team checked in with participants daily over WhatsApp for at least the first two days after enrollment to ensure they were equipped for the medication abortion process if they chose to proceed. Research team members who provided support to participants throughout the study were of Venezuelan origin, native Spanish speakers, and were trained and experienced in providing abortion accompaniment services. Participants were asked to indicate when their abortion was complete by filling out an in-app checklist within fourteen days of finishing the medication regimen and also by contacting the research team within this timeframe. Those who required additional resources or direct medical care were referred to appropriate external organizations.

When participants reported completion of a medication abortion to the study team, they were sent the quantitative research survey link over WhatsApp to be completed on SurveyMonkey. The anonymized survey included questions regarding the participant's basic demographic information, abortion and post-abortion contraception process, and experience using Aya Contigo, including how the mobile application impacted feelings of confidence, preparedness and support and its usability using a validated System Usability Scale (SUS) (37). The SUS questions were slightly adapted to fit the context of a mobile app for self-managed abortion (e.g., “I think that I would like to use this system frequently” was adapted to “If I imagine I was redoing the process, I would use Aya again”). The adapted SUS is available in Supplement B: Adapted System Usability Scale (SUS).

Those who opted to participate in the qualitative interview provided additional written informed consent after discussing the study procedures with a local researcher. Verbal consent was obtained to audio-record the interviews for transcription purposes and both parties confirmed that they had access to a private space with no one else present. Participants were scheduled for a 45–60-min virtual phone interview with a trained research team member. The interviewers asked in-depth questions about participants’ experiences with Aya Contigo and the abortion process. No repeat interviews took place. Participants were compensated $10 USD for completion of the online survey and received an additional $10 USD for the telephone interview. This incentive was transferred to participants over WhatsApp through a Venezuelan partner organization.

The recruitment target for the quantitative survey was set at 15 participants or greater, given that this descriptive data was collected for exploratory purposes and coupled with rich qualitative interview data. We sought to better understand the experiences of people seeking abortion within Venezuela's sociopolitical context, where the voices and narratives of pregnant people have been repressed for decades. In recent years, it has been exceedingly difficult to conduct research around sexual and reproductive health given governmental restrictions on data collection and reporting (3, 5, 38). Therefore, we deemed that even a small sample size would offer important insights on a historically under-represented population. The relatively small sample size target was set acknowledging that the survey sample cannot speak to the experiences of all people in Venezuela seeking abortion care. Taken together, the unique study population, the descriptive aim of our quantitative data, and its pairing with qualitative data justified a smaller survey sample size (39).

The recruitment target for the in-depth interviews was initially set flexibly, based on the concept of information power, a model introduced by Malterud and colleagues in 2016 (40). The study's aims and underlying theory, along with the specificity of experiences among included participants, the strength of interview dialogues, and our analysis framework were considered throughout the study period in order to determine an adequate sample size (40). We applied a human-centred design theory and defined a relatively narrow primary aim focused on evaluating Aya Contigo as an abortion and contraceptive care tool. The initial participant interviews elicited rich dialogue, and the restriction of participants to pregnant people seeking abortion amidst Venezuela's humanitarian crisis reflected a specific population with many common experiences. Taken together, our study design and participant characteristics increased the information power which enabled rich data from a relatively small sample. We deemed that a sample of 10–15 participants would yield sufficient information power.

### Data analysis

Descriptive analysis of the online survey was done in SPSS. Each outcome was reported as a mean (standard deviation) for continuous variables and count (%) for categorical variables.

The qualitative interviews were recorded, de-identified, transcribed in Spanish, and then translated into English for analysis. The transcribed data was thematically analyzed in NVivo 11. Two researchers trained in qualitative analysis methods, RG and KC, analyzed several transcripts to identify themes and to create codes and sub codes, organized into categories. This coding framework was applied to all transcripts by both researchers and modified through an iterative process.

Divergent participant views or opinions were reported as part of the results.

### Ethical considerations

The study received ethics approval from the Allendale Institutional Review Board. Participants were recruited passively to try and avoid any feelings of pressure to consent. Only basic, non-identifying, demographic information was collected to ensure participant confidentiality. Participants were informed throughout the study that they could withdraw at any time and could choose not to answer any questions on the online survey or telephone interview. If participants required support or care beyond the capabilities of the research team, they were referred to established trusted organizations in Venezuela or internationally, including Women Help Women, Safe2Choose, and Women on Web.

## Results

### Process overview

The context surrounding abortion in Venezuela greatly affected the implementation of the study. Recruitment into the study was initially challenging, as partner organizations that had planned to engage with potential participants faced increasing risks of government reprisal. The online recruitment ads targeted with relevant demographics were not effective, possibly due to lack of reach and/or trust. In order to navigate the increasingly challenging situation, the study team developed a Whatsapp chat line to recruit and connect with participants privately. Initially, this resource acted as a help desk to troubleshoot technical issues, but as participants vocalized a desire for additional support, the study team provided virtual personal support over Whatsapp.

The study also faced implementation difficulties due to participants’ lack of access to abortion medication or sufficient medication. Further, the guidelines of some local SRH support services differed from the WHO guidelines on medication abortion. It is important to acknowledge that these organizations offer safe and trusted means of SRH support to people in Venezuela. The Aya Contigo research team worked alongside other community allies in order to offer clarity around medication abortion guidelines. Despite the challenges faced, the study was able to continue thanks to the support of the local reproductive rights community and the trust that was built through the formation of long-term relationships with local stakeholders.

### Quantitative survey results

A total of 40 participants were recruited into the study between April and June 2021. Seventeen (42.5%) completed the online survey via SurveyMonkey. Table 1 displays the demographic characteristics of participants who completed the survey. All participants identified as women, with age ranging from 19 to 38 and a mean of 28 (SD 5.55). All women had at least completed some secondary education and 88.2% (15/17) reported at least some university training. While 70.6% (12/17) reported that they were actively employed, income was widely spread with 23.5% (4/17) reporting no income, 23.5% (4/17) earning minimum wage, 35.3% (6/17) earning double the minimum wage or more, and 17.6% (3/17) declining to respond. More than half of participants (9/17; 52.9%) lived in the Capital District or state of Miranda, while the others were spread across Barinas, Carabobo, La Guairá, Portuguesa, Yaracuy, and Zulia.

**Table 1:** Baseline demographics.

Total *n* = 17	*N* (%)
Gender	
Woman	17 (100)
Age	
≤20	2 (11.8)
21–25	3 (17.7)
26–30	6 (35.3)
31–35	4 (23.5)
>35	2 (11.8)
State	
Barinas	1 (5.9)
Capital District	4 (23.5)
Carabobo	1 (5.9)
La Guaira	1 (5.9)
Miranda	5 (29.4)
Portuguesa	1 (5.9)
Yaracuy	1 (5.9)
Zulia	1 (5.9)
Other	1 (5.9)
Highest level of education	
Some secondary education	1 (5.9)
Secondary education complete	1 (5.9)
Some university education	6 (35.3)
University complete	9 (52.9)
Employment	
Employed full-time	6 (35.3)
Employed part-time	2 (11.8)
Self-employed	4 (23.5)
Unemployed—looking for work	4 (23.5)
Unemployed—not looking for work	1 (5.9)
Income	
Minimum wage	4 (23.5)
Double minimum wage	3 (17.7)
More than double minimum wage	3 (17.7)
No income	4 (23.5)
No answer	3 (17.7)

The medication abortion process is detailed in Table 2. Gestational age at the time of initiating medication abortion ranged from four to twelve weeks, with 70.6% (12/17) of participants between six to nine weeks gestation. Mifepristone plus misoprostol was the regimen used by 76.5% (13/17) of participants while the remaining 23.5% (4/17) took misoprostol alone. When compared with a regular menstrual period, 76.5% (13/17) of women reported that their bleeding was heavier and 88.2% (15/17) experienced more severe pain during the abortion process. The most common side effects reported were diarrhea (64.7%; 11/17), dizziness (64.7%; 11/17) and nausea (52.9%; 9/17).

**Table 2:** Self-Managed medication abortion.

Total *n* = 17	N (%)
Gestational age (weeks) at time of abortion	
≤5	3 (17.7)
6–9	12 (70.6)
10–12	2 (11.8)
Abortion medication regimen	
Misoprostol only	4 (23.5)
Misoprostol and Mifepristone	13 (76.5)
Doses of misoprostol	
1	5 (29.4)
2–4	10 (58.8)
≥5	2 (11.8)
Symptom severity	
Bleeding compared with menstrual period	
*Lighter bleeding*	2 (11.8)
*Similar to normal*	2 (11.8)
*Heavier bleeding*	7 (41.2)
*Much heavier bleeding*	6 (35.3)
Pain compared with menstrual period	
*Less than normal*	1 (5.9)
*Similar to normal*	1 (5.9)
*Stronger*	5 (29.4)
*Much Stronger*	10 (58.8)
Side effects^a^	
Yes to any side effects	15/17 (88.2)
*Nausea*	9 (52.9)
*Subjective Fever*	5 (29.4)
*Diarrhea*	11 (64.7)
*Vomiting*	6 (35.3)
*Dizziness*	11 (64.7)
*Itchiness*	1 (5.9)
*Diaphoresis*	4 (23.5)
Abortion completion^b^	
Complete with no additional intervention	14 (82.4)
Complete with additional medication	1 (5.9)
Complete after surgical abortion	2 (11.8)
Time to pregnancy expulsion	
<12 h	10 (58.8)
36–48 h	1 (5.9)
Unsure or unaware of expulsion	6 (35.3)
Method(s) used to determine completion of abortion^a^	
Saw products expel	8 (47.1)
Aya Contigo checklist	9 (52.9)
Symptoms stopped	11 (64.7)
Counsellor or accompanier	3 (17.6)
Sought medical attention/ultrasound	5 (29.4)

^a^ Participants could select more than one answer.

^b^ Some participants completed the abortion completion survey too early in their course, and others did not complete this survey, so abortion completion was determined based on the study team's follow-up with participants 10 days after completing the regimen.

Abortion completion was assessed both through the survey results and based on communications with the study team via WhatsApp. After taking their medication regimen with the guidance of Aya Contigo and the virtual support chat, 82.4% of women (14/17) completed abortion without additional intervention. Based on self-assessment, 64.7% (11/17) reported that they knew when the pregnancy was expelled and 52.9% (9/17) utilized the Aya Contigo checklist for completed abortion. Five (29.4%) participants sought medical care to determine if the abortion was complete; one required additional medication to complete the abortion, two underwent surgical abortion, and the remaining two were reassured that the abortion was complete. All participants eventually completed abortion with no significant complications reported.

In the post-abortion stage, 58.8% (10/17) of participants were interested in contraceptive options (Table 3). Oral contraceptive pills and implant devices were the most desirable options. However, only two women were able to access contraception by the time of survey with both having obtained condoms. The most frequently reported barriers to obtaining contraception were cost (40.0%; 4/10) and insufficient time to obtain their preferred contraceptive option (30.0%; 3/10).

**Table 3:** Contraceptive uptake and methods.

	N (%)
Sought contraception	
Yes	10/17 (58.8)
Preferred contraceptive method(s) for those interested^a^	
Oral contraceptive pill	6/10 (60.0)
Implant	6/10 (60.0)
Intrauterine device	3/10 (30.0)
Depot Injection	2/10 (20.0)
Condom	2/10 (20.0)
Method obtained for those interested	
Condom	2/10 (20.0)
Unable to post-abortion access	8/10 (80.0)
Barriers preventing access	
– Cost	4
– Unsure where to find	1
– Still waiting for preferred option to arrive	3
Source(s) used to select preferred contraceptive^a^	
Aya Contigo	8/10 (80.0)
Friend or relative	3/10 (30.0)
Healthcare provider	5/10 (50.0)
Internet	3/10 (30.0)

^a^ Participants could select more than one answer.

Participants responded to Likert scale questions focused on their experiences with Aya Contigo as a medication abortion support tool (Table 4). All participants expressed that the mobile application supported them during the abortion process, with 52.9% (9/17) feeling “very supported” and the remaining 47.0% (8/17) feeling “somewhat supported.” Overall, participants felt prepared for common abortion symptoms, with 87.5% (14/16) and 94.1% (16/17) “somewhat prepared” or “very prepared” for the respective pain and bleeding they experienced. All participants (17/17; 100%) reported that Aya Contigo contributed to their knowledge of the abortion process. For both determining abortion completion and choosing a contraceptive method with Aya Contigo, 41.2% (7/17) participants reported that they felt “very” confident, while 52.9% (9/17) reported that they were “somewhat” confident, and one participant reported “little” confidence. On the SUS, Aya Contigo scored 83.38 (out of 100), which corresponds to an “A” grade in the top 10% of scores (37).

**Table 4:** Experiences with Aya contigo.

Likert scale (total *n* = 17)	Not at all	A little	Somewhat	Very	Missing
*In general, how supported did Aya Contigo make you feel during your abortion process?*	–	–	8 (47.1)	9 (52.9)	0
*To what extent did Aya Contigo contribute to increasing your knowledge about the abortion process?*	–	–	1 (5.9)	16 (94.1)	0
*How confident were you determining your gestational age with Aya Contigo?*	–	3 (17.6)	6 (35.3)	8 (47.1)	0
*How helpful was Aya Contigo in determining you were taking the right kind of pills?*	–	1 (6.3)	5 (31.3)	10 (62.5)	1
*How confident were you using Aya Contigo to make sure you took the correct dose of pills at the correct time?*	–	1 (6.3)	4 (25.0)	11 (68.8)	1
*How prepared did Aya Contigo make you feel for the pain/cramping that you experienced?*	–	2 (12.5)	7 (43.8)	7 (43.8)	1
*How prepared did Aya Contigo make you feel for the bleeding that you experienced?*	–	1 (5.9)	10 (58.8)	6 (35.3)	0
*How confident were you in determining your abortion was complete with Aya Contigo?*	–	1 (5.9)	9 (52.9)	7 (41.2)	0
*To what extent did Aya Contigo contribute to increasing your knowledge about contraceptive methods?*	–	–	8 (50.0)	8 (50.0)	1
*How confident did you feel choosing a contraceptive method with Aya Contigo?*	–	1 (5.9)	9 (52.9)	7 (41.2)	0

### Qualitative results

Thirteen of the 17 participants who completed the survey completed the semi-structured phone interview after providing additional informed consent. The analysis of the interviews explored themes within three overarching categories: (1) abortion experience in Venezuela, (2) participant experiences with Aya Contigo, and (3) role of Aya Contigo within the existing ecosystem of safe abortion care in Venezuela. Seven sub-themes were identified iteratively during the coding process and are detailed in Table 5.

**Table 5:** Qualitative themes, organized by relevant codes and sub-codes.

1. **Abortion experience in Venezuela** a) *Local context impacts abortion experience and decision making* • Social, economic, humanitarian context in Venezuela ◦ Difficulty finding/accessing resources and seeking medical care ◦ Fear of legal retribution around abortion and associated services • Lack of personal support and perceived stigma • Strong desire for privacy and security with the abortion process b) *Accessing abortion pills markets presents numerous challenges* • Difficulty accessing mediation abortion regimens that are safe and trusted ◦ Access routes: clandestine/informal market vs. via trusted partners • High cost of mediation abortion can be prohibitive • Misinformation around type of pills and the correct dosing regimen 2. **Participant experiences wtih Aya Contigo** a) *Acceptability and comfort with Aya Contigo* • Aya Contigo is highly usable and offers appealing design features • User concerns ◦ Privacy and security ◦ App glitches b) *Aya Contigo can support self-managed medication abortion* • Supported preparedness and confidence around the abortion process • Steps of self-managed abortion with Aya Contigo ◦ Relatively straightforward self-assessment for eligibility ◦ Confusion around self-assessment for completion • Experience with the abortion process itself ◦ Increased readiness and knowledge around abortion symptoms and their management c) *People wanted contraceptive guidance after self-managed abortion* • Women interested in learning about contraceptive options with Aya Contigo • Desire to avoid an unexpected pregnancy after undergoing the abortion process • Access to contraception remains very difficult and expensive d) *Virtual chat support positively impacted the abortion experience* • Women wanted additional support and someone to ask for help • Added security and confidence with access to virtual support • Virtual accompaniment not the same as having someone in-person to provide support 3. **Role of Aya Contigo (the mobile app and virtual chat) in existing ecosystem** a) Aya Contigo can play an important role within the existing Venezuelan ecosystem of self-managed abortion and contraception care • Connecting women to other services • Filling a gap in terms of self-managed abortion support and information

### Abortion experience and decision making in the Venezuelan context

#### Legal context

When describing their perceptions of abortion, many participants discussed the implications of the legally restrictive setting in Venezuela. The fact that abortion is illegal in Venezuela in almost all circumstances increased the level of fear and anxiety associated with choosing to pursue an abortion. In this context, people described difficulty seeking out support due to the risk of penalization. Participants were often wary to disclose their interest in abortion or history of abortion to community members, including healthcare providers. As one participant explained:

“[The legal context] influenced a lot because … everybody is afraid. In fact, in December they imprisoned [an activist] who helped a girl who had been raped… from then on, many people never again gave those pills and so on.”—Participant 6

This quote refers to an event that occurred a few months prior to the study period, where a Venezuelan feminist advocate and educator was arrested and charged after helping a 13-year-old girl to end a pregnancy after being raped (41). As a result of this widely publicized arrest, many feminist organizations and activists halted their abortion support services, including the provision of medication for self-managed abortion. Many agreed that the legal restrictions had limited access to resources for abortion:

“I wish they could legalize abortion here in Venezuela…because I think there are many people who think the same as I do right now, and maybe they do not have the knowledge and do not know where to turn to, or where to ask, because they think it is illegal, so they do not ask, they do not do it, I think it is worse.”—Participant 29

In this legally-repressive environment, participants did not have a clear means of asking for help or seeking support. All participants faced significant barriers in accessing medical, psychological, and educational services. As a result, most resorted to finding information online, but were unsure which sites they could trust.

#### Humanitarian/economic context

The economic instability and broader humanitarian crisis in Venezuela played an important role in participants’ decisions to seek abortion. Many felt that they could not provide for a child, or that a child would not be able to thrive in their current environment. This sentiment was captured by a participant who considered the multifaceted issues that many people in the country continue to face:

“It is such a complicated situation [in Venezuela] that [makes having] a child a bit more uphill. Because of the economic situation, the social situation, the political situation..there are many difficulties, including the health system and the transportation situation. It is a level of complexity so high that … you could not even fantasize about everything that happens here.”—Participant 34

As articulated in the above quote, many felt that the challenges in their day-to-day lives in Venezuela would be unimaginable to people elsewhere. Many of these challenges were exacerbated by hyperinflation and relatively low incomes, which often made basic services difficult to afford. The COVID-19 pandemic exacerbated the economic difficulties of all participants; several had lost their means of employment and income. All participants felt they were struggling financially, including those who remained employed and earned above the average income level. Some acknowledged that despite their difficulties, others had been hit even harder by the economic crisis:

“My economic situation is very vulnerable right now, really, it is very critical. But there are women who are worse off than me.”—Participant 25

As the protracted economic collapse in Venezuela reduced the number and capacity of healthcare provision services, many participants described that even basic health services were extremely limited. Private care was prohibitively expensive and public clinics had long wait times. In this context, participants also reported difficulty accessing and affording sexual health services. As one participant described, some contraceptive options were no longer available, and costs associated with accessing contraceptives continued to rise:

“Contraceptives as they used to come, they don't come anymore…lately the consultations are very expensive…to get the pill you have to go for a consultation and the consultation has gone up to $20 [USD].”—Participant 18

Overall, participants described that they did not feel equipped to support a child, yet they were often unable to prevent pregnancies with reliable contraceptive options. In this fraught ecosystem, many were stripped of their reproductive health autonomy while struggling to provide for themselves and their families.

#### Personal context

In addition to the socioeconomic and legal context in Venezuela, participants’ personal lives and relationships impacted their experience with abortion. All restricted the disclosure of their abortion to a select few, if any, others:

“Only three people knew, my husband, my mom and me.”—Participant 38

For many participants, disclosure to one or two trustworthy confidants acted as a means of support during a difficult time. All pregnant people interviewed expressed concern that others might find out, and wanted their abortions to occur as discreetly as possible. Some reported that this was due to the legal risks while others perceived a social stigma around abortion. As one participant described, abortions often happen covertly due to sociocultural disapproval:

“[Abortion] is a taboo, people are very religious, moralistic, there are many prejudices, the subject is taboo. So, you have to do everything on the sly, it's like that. So, it is very difficult to do that.”—Participant 3

The secrecy surrounding the abortion process led many to feel alone, even if they had disclosed this to a friend or family member. Several participants highlighted the contrast between the isolation of the abortion process and the community support received during a continuing pregnancy. In addition, many reported that the stigma surrounding abortion created guilt and anxiety about their decision. Despite this, most were resolved that abortion was a necessary decision. The conflicting emotions that many participants experienced were summarized by one participant:

“I felt very ugly. I felt a lot of guilt, that's what it was. Still. I think it's something I have to get over…in the end, that was what I wanted, and it was the best decision I could make.”—Participant 3

When participants decided to go through with an abortion, the emotional distress that many experienced was often heightened by cultural perceptions of abortion as immoral or wrong. There was a shared motivation among participants to keep the process secret for both legal and cultural reasons, which prevented several participants from seeking additional support.

### Abortion pills are difficult to access

The Venezuelan legal restrictions, economic precarity, and significant gaps in healthcare provision posed challenges when participants attempted to obtain medication abortion pills. Some said that misoprostol was available in local pharmacies, but the medication requires a prescription, and cannot be prescribed for abortion. Given that abortion medications are not easily obtained from healthcare institutions, participants reported that they had searched for medication through clandestine online marketplaces or by word of mouth. Some were connected with pharmacies that sold prescription abortion medication covertly, as one participant explained:

“…I looked for them on the internet, because that's the only way here to get them, but a friend of mine who is a doctor recommended me to a pharmacy… it gave me more security to buy them there, than to buy them from any person in the street, you don't know where those pills come from.”—Participant 29

While some participants felt a sense of security that their medications were dispensed by organizations affiliated with medical pharmacies, others obtained their abortion medications from non-regulated sources. When medications were purchased online or through other clandestine means, participants were concerned about the safety of the pills. Some participants had heard rumors or knew someone that had been harmed by abortion pills acquired in this manner:

“…they also said that it could be fake or that some were modified and that many people had died because of that.”—Participant 6

The interviews reflected the precarity of buying abortion pills from the unregulated market in Venezuela. In addition to causing potential harm, many participants were concerned that the pills would be ineffective in terminating their pregnancies.

Many participants also faced challenges related to the cost of abortion pills: participants reported that prices per misoprostol pill ranged from $3–$35 USD, most commonly around $20 USD. Given that a proper regimen consists of several pills, the total cost often exceeds $100 USD. Some participants were able to purchase enough pills, but for others, the cost prohibited the purchase of a full regimen. As one participant explained,

“…Each pill costs me 35[US] dollars, imagine 12 pills and as I said I am low income.”—Participant 37

The high expense of abortion pills added to the financial concerns of each participant. While all participants were able to acquire some medication, those who could not afford a full regimen were at risk of an ineffective termination.

### Acceptability and comfort with Aya Contigo

All participants reported feeling comfortable using Aya Contigo and trusted the information that the app provided. The educational tools and checklists for self-managed abortion were described as detailed and clear. Every participant reported that they would recommend Aya Contigo to others in need of abortion care. As one participant shared:

“If a person I know needs that help I will recommend [Aya Contigo], of course. You are very safe, you give a lot of security, and all the questions are answered.”—Participant 6

Many participants similarly reported that they could buy-in to Aya Contigo because of its security and discretion in the self-managed abortion process. They appreciated that its name was vague and would not attract attention, and that the application could be easily deleted with no remaining data stored on their personal devices. However, some participants wished that a login passcode was required in order to open Aya Contigo for additional digital security:

“The application gave me security…As an application, perhaps the way to log in with a code, would [increase security].”—Participant 2

Some participants were fearful that a friend or family member would find the application on their phone and discover that they were pursuing abortion. In contrast, others felt their phones were already private, and that the only person who might see the app (e.g., a partner) was already aware they were having an abortion. In the context of a highly restricted legal and social setting, the clear portrayal of relevant abortion-specific information, and the inclusion of security features within Aya Contigo's design were key factors for its acceptability among participants.

Participants found that Aya Contigo was user-friendly and aesthetically pleasing; the overall design and color scheme was described as attractive, feminine, and calming. Participants were all Spanish-speaking and were able to understand the language used in the application. Several participants found that the images embedded in the app helped clarify some of the medical information included. As one participant explained:

“Maybe with the word you don't understand … but then with the image you associate the issue and you understand it.”—Participant 6

A few participants suggested including more illustrations for this reason. Despite the need to include some medical terminology, the information provided in the application was frequently described as “easy” to navigate and understand.

There were some usability aspects of the application that led to initial difficulty for some participants, though most were resolved with troubleshooting or small adjustments. For example, the app froze for a few participants, but this was resolved with support from the research team on how to clear and re-download it. The ability to solve problems in real-time with the study team members was described as reassuring by some participants.

### Aya Contigo can support self-managed abortion

Participants overall expressed that Aya Contigo had created a feasible means to facilitate self-managed abortion, by including comprehensive self-assessment tools for medication abortion eligibility, the abortion process itself, and the determination of abortion completion. As participants describe of their overall experience with Aya Contigo:

“I followed the steps just as the application told me, even though I have friends around me who suddenly told me that I had to do the procedure in a different way…I felt that what I was doing was right, that I was following the steps as they told me there, I didn't need to look on the internet or anything like that… And it was fine, I felt pretty confident.”—Participant 10

I felt comfortable … because I felt that [Aya Contigo] was there, “look, it's your turn now, the time has come,” and I really liked it.”—Participant 34

The resources and information provided by Aya Contigo supported participant confidence and preparedness for the medication abortion process. Some participants used Aya Contigo as their sole source of information, and most felt it successfully guided them.

The initial self-assessment for medication abortion eligibility was straightforward for most participants and included an assessment of gestational age based on menstrual period. Most participants had already obtained an obstetrical ultrasound, and there were a few cases where the application's calculation differed from the dating ultrasound. Most participants continued to use the dates based on the ultrasound but still appreciated the idea of the gestational age calculation within the app*.* Once eligibility to proceed with self-managed abortion was confirmed, many expressed that Aya Contigo was useful in preparing them for the medication regimen and abortion process itself. Some were confused about the dosing schedule because it differed from information that they had received from other sources, or because they had already taken some abortion pills obtained from the informal market. In the few cases where additional clarification was required, participants communicated with study team members through the WhatsApp chat which was effective in resolving their queries.

Participants expressed that Aya Contigo was useful for managing symptoms of the abortion. The ability to review the difference between normal symptoms and warning signs that would require medical attention in the app allowed many to identify that their symptoms were to be expected. In order to tailor the information to the individual user, the application provided different information on possible symptoms and side effects depending on whether the participant was taking the mifepristone plus misoprostol regimen or the misoprostol regimen alone. By knowing to anticipate symptoms such as moderate to heavy bleeding and cramping, many felt reassured. However, some participants had hoped the application would provide more information about other known side effects:

“Obviously the process of diarrhea is normal…But it would be good to be alerted so that there is no doubt about it…”—Participant 34

Each person experiences the abortion process uniquely. Because of this, the app's description of symptoms and the timeline of the abortion process aligned more closely with some participants’ experiences than others. Overall, participants reported feeling prepared for the abortion process when using Aya Contigo, especially around common symptoms of pain and bleeding.

After the medication regimen was complete, participants were asked to complete Aya Contigo's checklist for abortion completion. The self-assessment for abortion completion was generally found to be useful but was more complicated than the other steps of the process. Based on their responses, many participants were alerted that they could still be pregnant (often because they responded “yes” to questions about ongoing symptoms such as vaginal bleeding). This created anxiety and confusion, as they had otherwise believed that the abortion had been successful. This was likely because they completed the assessment too early in the process, instead of the 7–14 days recommended in the app. One participant explains that this could have been made clearer:

“This [step] should be very specific, whether it is going to be filled in one or two weeks or after 10 days, because a week's difference is quite a lot for this process, so it should be available only after a certain time..”—Participant 30

In order to reduce the risks of confusion and anxiety that the pregnancy may not have been terminated, one participant suggested that the checklist only become available 7 days after completion of the medication regimen. They also recommended that the reporting of minor symptoms in the app's completion checklist should not immediately trigger the app to raise the concern of incomplete abortion. A few participants had no concerns with the completion self-assessment and reported that it increased their confidence that the abortion was complete*:*

“The next day what I did was complete an [assessment] asking me if my abortion was completed. There I put all my experience, all the options that were integrated by what I had gone through. It made me feel a little more confident about what I had done, and everything went well.”—Participant 3

Aya Contigo offered support for individuals undergoing the process of self-managed medication abortion. Several participants utilized the abortion completion checklist to determine whether their abortion was complete. This was viewed as useful to some, while others feared they might still be pregnant due to ongoing symptoms such as vaginal bleeding. This led participants to seek additional advice from the study team or a medical provider.

### People wanted contraceptive guidance after self-managed abortion

The contraceptive portion of the app was described as helpful and comprehensive, enabling participants to make informed decisions about their reproductive health. All of the participants thought that the section was particularly useful in the post-abortion period, as one woman highlighted:

“I think it is necessary to have [the section on contraception], and it was good to see it because after you go through [an abortion], you think, well, I hope I don't have to go through something like this again, so you have to be more careful, be aware of or evaluate other contraceptive methods that work well for the body, so it is necessary to have that information.”– Participant 29

Learning about contraception through Aya Contigo reinforced and nuanced participants’ existing knowledge about the different types of birth control options, including their effectiveness and side effects. After using Aya Contigo's contraceptive tool, several participants identified the option that would best suit them, most commonly expressing interest in a long-acting reversible contraceptive. However, some were unsure that they would be able to access their preferred option. Some participants also had ongoing misconceptions or fears about certain contraceptive methods. For example, one woman reported:

“I [knew] one who used [a copper IUD], I don't know if she didn't take care of herself, didn't go to the doctor, and that caused her to get a tumor down there and as a result she died, so I don't like them.”—Participant 18

For the few with specific preconceived negative impressions, it was unclear if the application's contraception tool shifted their views. Overall, despite the legitimate concern that contraceptive options may be difficult to access, Aya Contigo's contraceptive tool enabled participants to feel prepared and confident to pursue an evidence-based method for the prevention of future pregnancy if they wished.

### Pivoting to virtual chat support positively impacted the abortion experience

A virtual help desk over WhatsApp was created to facilitate study recruitment and research-related consent processes. This chat enabled the research team to communicate with participants over a familiar platform in a safeguarded manner. When participants were enrolled and began undergoing the medication abortion process, it became clear that they required support beyond Aya Contigo alone. While the WhatsApp chat was initially created as a research help desk, this communication channel became a component of the self-managed abortion process itself. Study team members staffed the line 24/7 and responded to questions and concerns throughout the process. The team also provided emotional support when participants reached out and acknowledged that they were struggling.

Once established, real-time virtual Whatsapp chat was considered valuable by all participants. Some found the way Aya Contigo and the chat support guided them through each step was akin to having a counsellor helping them with the abortion process, as one participant described of the app:

“I had a support, a friend, a trusted person.”—Participant 2

The chat was reassuring and calming for many participants. The ability to confirm information in the app alongside the presence of someone checking in with them made them feel more supported during an otherwise isolating process. All participants felt more confident knowing there was a real person available and attentive to them, highlighted by one participant:

“I was always on the app and talking to someone … through WhatsApp, I didn't feel uncomfortable with the app because anything that appeared on the app that generated any kind of concern I could then solve it through WhatsApp…the responses so in the moment, so active, so present…really it was a very good experience.”—Participant 34

The trust and support provided by the research team gave participants more confidence in their ability to self-manage the abortion process. Several participants noted that they would have preferred the chat to exist in the app itself instead of over Whatsapp.

### Aya Contigo can play an important role within the existing Venezuelan SRH ecosystem

At the time of the pilot study, there were already existing humanitarian organizations in Venezuela working to try and improve access to reproductive healthcare and abortion services. Given the country's oppressive environment, many participants reported that they did not know how to access these services. Some participants noted that while they remained in contact with Aya Contigo's team, other harm-reduction organizations could not stay in regular communication with them:

“[Aya Contigo team] were a support because later they stopped answering me from [local organizations], and I really kept communicating with you… and it was really good.”—Participant 6

The ongoing communication that Aya Contigo's team could provide was an important component of the abortion care experience. By establishing trust through this communication channel, participants could seek additional support and ask questions to the team when needed. Participants appreciated that the study team offered referrals to a local sexual and reproductive health clinic that offered contraceptive resources.

For some participants, Aya Contigo was viewed as an additional resource and support that improved their abortion experience. For others, the information, support, and referrals provided by the Aya Contigo team were critical to feeling they had the resources and confidence to successfully complete the abortion process:

“It's just that if I hadn't known [Aya Contigo], I wouldn't have known that person either. Because without the communication with you, how would I also have met those [other trusted source] people? How would I have gone? I would not have met them, and they would not have helped me. That is why I tell you that you were a fundamental tool.” -Participant 38

Overall, participants valued that study participation enabled them to connect with other sexual and reproductive organizations operating locally. These connections usually happened outside of the app itself; most participants did not use the recommended resources section as it listed organizations outside of Venezuela. Participants reported that they would prefer if local organizations or doctors were listed in the app, although many understood that this would be challenging to implement given the legally restrictive setting.

## Discussion

Aya Contigo is the first digital application designed to support medication abortion and contraceptive care for pregnant people in Venezuela, where sexual and reproductive health rights are heavily repressed and maternal mortality rates are rising. Building on the ground-breaking work of activist and feminist organizations in the region, Aya Contigo shows promise as an intervention to support self-managed medication abortion and post-abortion care. The digital app's informational guides and real-time virtual accompaniment service helped participants feel prepared, confident, and supported during the abortion process. Aya Contigo also connected participants with local sexual and reproductive health resources and enabled individuals to select a contraceptive option post-abortion. The digital intervention can bridge existing SRH services by expanding access to evidence-based information and accompaniment services for self-managed abortion in Venezuela.

Aya Contigo's informational guides for medication abortion were designed to provide pregnant people with a clear understanding of the process. The interactive abortion eligibility checklist and the medication abortion steps were highly valued by participants and helped them feel prepared. Guidelines suggest that medication abortion pain can be severe and that people undergoing abortion should have adequate resources to reduce suffering (42). While the pain symptoms associated with medication abortion have historically been minimized by providers (43–45), the digital tool helped participants anticipate pain and bleeding symptoms. While most study participants reported that the pain and bleeding associated with medication abortion was more severe than a regular menstrual period, they often felt confident in managing their symptoms with the support of Aya Contigo.

In addition to the application's informational features, the virtual accompaniment service was a key factor in the intervention's uptake and acceptability among participants. While Aya Contigo was initially developed as an evidence-based information source for people seeking self-managed abortion, the need for individualized support became evident early on in the study. Aya Contigo's in-app abortion guide was then coupled with a real-time virtual chat over Whatsapp with a study team member trained in abortion accompaniment. Personal accompaniment services have been shown to increase the efficacy of self-managed medication abortions in legally-restrictive settings (46, 47). The virtual accompaniment service provided guidance around health-related questions and assistance with any unique challenges encountered throughout the abortion process. Virtual accompaniers offered important emotional support, though some expressed that a digital tool cannot replace in-person support. While digital health solutions are not a panacea, Aya Contigo's model shows promise as an intervention for people seeking abortion in under-resourced and restrictive settings. Since study completion, the virtual chat support function has been embedded within the app itself to facilitate streamlined communication with a team of trained accompaniers.

There were some important challenges encountered during the self-managed abortion process, which participants described in the interviews. Almost all participants had difficulty accessing and affording safe abortion medications, which is a known and pervasive problem within Venezuela (7, 9). While all participants had a complete abortion, three required further intervention, most likely because they could not initially obtain a full abortion medication regimen. By partnering with trusted organizational partners, Aya Contigo provided participants with information about where they could access essential resources and further healthcare if needed.

The Aya Contigo checklist for abortion completion was created based on evidence that patient post-abortion checklists are accurate and comparable to in-clinic assessment (48). However, some participants were inappropriately alerted that they may still be pregnant after completing the in-app checklist, a concern that has been reported by other research evaluating self-managed abortion (49). In order to reconcile this, participants clarified their concerns and symptoms over the real-time chat with trained accompaniers. Based on participant feedback, the app now prompts participants to check-in with the virtual care team or a healthcare provider if there are any flagged ongoing symptoms or concerns.

Cybersecurity is an area of growing concern within the FemTech space, as protecting user privacy and security is of utmost importance when offering abortion services in legally-restrictive settings. Participants appreciated that Aya Contigo provided them with an anonymized and discreet option for self-managed abortion. Recognizing the risks of criminalization in Venezuela, participants seeking abortion suggested that the app include additional safeguards. In order to minimize the risk of participant data breaches from unwanted or punitive third parties (27, 50, 51), the virtual chat component was embedded into the app itself. All users receive information on how to permanently delete information from their personal devices, and back-end data security policies are regularly reviewed to ensure maximum encryption. The ability to evolve with the legal and technological landscape will be essential to protect the security and privacy of Aya Contigo users.

## Limitations

The study was conducted within an active humanitarian crisis, where citizens and research team members experienced economic, infrastructural, and socio-legal challenges on a daily basis during the study period. This context limited participant recruitment and retention in research, particularly because the study focused on abortion, which is heavily restricted and criminalized in Venezuela. As a result of these constraints, the study has important limitations in its scope and size. There was high loss to follow-up throughout the study recruitment period, which has been similarly reported in other studies evaluating self-managed abortion (49).We completed recruitment and data collection on a small convenience sample of 17 participants, which limits the study's generalizability. Participants’ experiences with self-managed medication abortion as guided by Aya Contigo may not represent the experiences of the wider Venezuelan population seeking sexual and reproductive health care. All participants identified as women, and therefore the unique narratives of trans and non-binary people experiencing pregnancy are not captured in the data. Further, the majority of participants lived in urban settings and reported high levels of education which is not representative of the national average. The fact that this service was viewed as useful even among those with higher resources than average suggests that this intervention may be useful to those in greatest need.

In line with current data on the safety and efficacy of self-managed medication abortion, the study restricted enrollment to those within the first trimester of pregnancy. As a result, the possible benefits of this digital guide and accompaniment service for medication abortion are limited to people in early pregnancy. In an effort to support those beyond 12 weeks of gestation, the Aya Contigo application provided information on trusted local resources and healthcare facilities that offered reproductive health services.

Data was collected in Spanish by research team members native to Venezuela, but this data was translated to English for analysis, which has the potential to lose some of the original meaning. The study relies on self-reported outcome data, which is valuable for understanding participants’ experiences but cannot be corroborated. Qualitative and quantitative data were collected at a single time point from participants, most often within the 2-week period following self-managed abortion, which prevents the reporting of participant experiences months or years after the abortion. While the study benefitted from utilizing an adapted SUS scale and garnered rich qualitative data, the sample size was ultimately too small to make definitive conclusions around the application's acceptability and feasibility in Venezuela.

### Implications

The results of this pilot study have informed the iterative development of Aya Contigo and its affiliated services for people seeking abortion and contraceptive care in Venezuela. In 2022, Aya Contigo was released to the general population in Venezuela and early data reveals significant uptake among people interested in self-managed medication abortion (8). The World Health Organization selected Aya Contigo as one of five key interventions in the global abortion care movement as part of International Safe Abortion Day 2023 (52). Beyond the application's introduction in Venezuela, this study offers insights into digital health tools for abortion care in legally-restrictive settings. Our results echo the need for localized, human-centered design in the development of mobile applications and other interventions to effectively meet the needs of users. This model can act as a guide for other abortion and SRH-focused digital health interventions, particularly within humanitarian or restrictive settings.

## Conclusions

This mixed-methods pilot study demonstrates that the Aya Contigo mobile application may support pregnant people seeking medication abortion and post-abortion contraceptive services in Venezuela. Participants valued the provision of evidence-based information, virtual accompaniment services, and locally-available sexual and reproductive health resources via the digital tool. Further research and interventions are needed to ensure that all pregnant people in Venezuela can access safe abortion and contraceptive resources.

## Data Availability

The datasets generated and/or analyzed during the current study are not publicly available as they contain sensitive information about structurally marginalized populations. Raw data supporting the conclusions of this article will be made available by the corresponding author, upon reasonable request.
