# Supplementary material for: Aya Contigo: evaluation of a digital intervention to support self-managed medication abortion in Venezuela
Source: Front Glob Womens Health. 2024 Oct 17;5:1401779. doi: 10.3389/fgwh.2024.1401779 (PMC11524962; doi:10.3389/fgwh.2024.1401779)
Supplement: Supplementary file 1 [file Datasheet1.docx]

**Supplement**

Supplement A: Abortion Completion Checklist

| If you took the abortion pills within the last 3 weeks, and experienced bleeding and cramping, let's assess how you are doing with this checklist. For all questions, users can respond either *yes* or *no.*   1. Did you have cramps after taking all the pills? 2. Did you have bleeding at least similar to your usual menstrual period after taking all the pills? 3. Did you have blood clots or tissue after taking all the pills? 4. Have pregnancy symptoms (breast tenderness, nausea or "morning sickness", frequent urination, exhaustion or tiredness) disappeared? 5. Do you think you are still pregnant, or do you still feel pregnant at this time? 6. Are you bleeding heavily today (more than 2 maxi pads in 1 hour for 2 hours at a time or blood clots that are larger than an orange)? 7. Do you have a fever today? 8. Do you have cramps or pain today? |
| --- |

Supplement B: Adapted System Usability Scale (SUS)

| Participants rated each question on a scale from 1 to 5, where 1 is ‘strongly disagree’ and 5 is ‘strongly agree.’   1. If I imagine I was redoing the process, I would use Aya again 2. I find Aya difficult to use 3. I thought Aya was easy to use 4. I think that I would need the support of a technical person to be able to use Aya 5. Aya guided me smoothly through the process and decision making 6. I thought there was too much inconsistency in Aya 7. I imagine that most people would learn to use Aya very quickly 8. I found Aya very cumbersome to use 9. I felt very confident using Aya 10. I needed to learn a lot of things before I could get going with Aya |
| --- |
